# Elastic stiffness coefficients of thiourea from thermal diffuse scattering

**DOI:** 10.1107/S1600576720016039

**Published:** 2021-02-01

**Authors:** Julia Büscher, Alessandro Mirone, Michał Stękiel, Dominik Spahr, Wolfgang Morgenroth, Eiken Haussühl, Victor Milman, Alexei Bosak, Oleh Ivashko, Martin von Zimmermann, Ann-Christin Dippel, Björn Winkler

**Affiliations:** a Institute of Geosciences, Goethe University Frankfurt, Altenhöferallee 1, Frankfurt am Main, Germany; b Deutsches Elektronen-Synchrotron DESY, Notkestrasse 85, Hamburg, Germany; c European Synchrotron Radiation Facility, 71 avenue des Martyrs, Grenoble, France; d Dassault Systèmes BIOVIA, Cambridge, United Kingdom

**Keywords:** thermal diffuse scattering, elastic stiffness, thiourea

## Abstract

The complete elastic stiffness tensor of thiourea has been determined from thermal diffuse scattering (TDS) using high-energy photons (100 keV). Comparison with earlier data confirms a very good agreement of the tensor coefficients. In contrast with established methods to obtain elastic stiffness coefficients (*e.g.* Brillouin spectroscopy, inelastic X-ray or neutron scattering, ultrasound spectroscopy), their determination from TDS is faster, does not require large samples or intricate sample preparation, and is applicable to opaque crystals. Using high-energy photons extends the applicability of the TDS-based approach to organic compounds which would suffer from radiation damage at lower photon energies.

## Abstract 2

The elastic stiffness coefficients of thiourea are determined from thermal diffuse scattering.

## 1. Introduction   

The elastic stiffness tensor *c* describes how stress relates to strain. It comprises at most 21 independent coefficients for a triclinic crystal, the *c*_*ij*_ coefficients (in Voigt notation), which are determined by the bonding system and the properties of the atoms (Fedorov, 1968 ▸). Knowledge of the complete tensor allows investigation of the anisotropy of the bonding chains and the derivation of numerous physical quantities, *e.g.* the velocity of sound waves, the bulk modulus and the Debye temperature. Several methods exist for determining the elastic stiffness coefficients of bulk single crystals but all of them have shortcomings restricting their applicability. Brillouin spectroscopy (Brüesch, 1986 ▸) can be employed only if the crystals are transparent, and the sample preparation is time consuming and complicated, especially for low-symmetry crystals. Determining the elasticity of opaque samples is only possible with Brillouin scattering for surface layers or thin films [see *e.g.* Speziale *et al.* (2014 ▸) or Sandercock (1982 ▸) for reviews]. The determination of the slopes of acoustic phonons by inelastic neutron scattering (INS) or inelastic X-ray scattering (IXS) (Krisch & Sette, 2017 ▸) requires very time-consuming measurements. Additionally, for INS, large samples (several cubic millimetres in size) are necessary, and for IXS, only a very few beamlines at synchrotron radiation facilities exist that allow the required high-resolution energy- and momentum-resolved measurements to be carried out. For ultrasound techniques, *e.g.* resonant ultrasound spectroscopy or plane-wave/parallel-plate ultrasound spectroscopy (Arbeck *et al.*, 2010 ▸), large samples with dimensions of a few to several millimetres are required, and the sample preparation is time consuming and can be difficult. In order to determine the elastic tensor from impulse-stimulated light scattering, the preparation of a number of differently oriented crystals (depending on symmetry) is necessary, and measurements of surface-wave velocities have to be carried out in different directions across the crystal. Hence this method is both time consuming and experimentally challenging (Waeselmann *et al.*, 2016 ▸).

In 1948, Olmer obtained information on the elastic behaviour of a cubic crystal from thermal diffuse scattering (TDS) for the first time (Olmer, 1948 ▸). Recently, Wehinger *et al.* (2017 ▸) were able to determine the complete elastic stiffness tensor of trigonal calcite and cubic magnesium oxide from TDS. This approach has several advantages compared with the techniques mentioned above. The experimental setup is straightforward because the TDS data can be obtained from a comparatively fast (a few hours) single-crystal X-ray diffraction experiment. Additionally, it allows temperature-dependent measurements. Sample preparation is also straight­forward as, in contrast to many other methods, the crystals do not have to be cut and polished in specific orientations. If high photon energies are employed, a smooth sample surface is not necessary. The samples do not need to be transparent and small sample sizes (tens to hundreds of micrometres) are sufficient. Girard *et al.* (2019 ▸) demonstrated that this approach can be used to obtain the tensor of an orthorhombic crystal. The purpose of the present study is to expand this approach to organic materials, where sometimes only small crystals unsuitable for many other methods, *e.g.* ultrasound spectroscopy, are available.

In our preliminary TDS experiments with 14 keV photons, we found that our organic samples suffered radiation damage after being exposed to the beam for just a few minutes. The damage resulted from a high photoelectric absorption coefficient. Consequently, we decided to explore measurements at much higher photon energies which are expected to reduce the absorption effect. Also, due to noticeable surface scattering in the preliminary experiments it was difficult to filter out the TDS. Experiments at higher energies would allow the sample volume to be increased to obtain a more favourable volume-to-surface ratio.

Only a few diffuse scattering studies using high-energy photons have been reported up to now. Gibaud *et al.* (1997 ▸) demonstrated that the combination of high-energy (60 keV) X-rays and an image-plate detector allowed measurements of diffuse scattering intensities with 120 s exposure times per image. They noted that their use of a 3 mm thick crystal significantly minimized the effect of diffraction at the crystal surface. Also, they pointed out that large area detectors intersect a nearly flat section of the Ewald sphere when using high-energy X-rays, which simplifies the data analysis. Later, Ramsteiner *et al.* (2009 ▸) showed that in such experiments the ratio of coherent scattering to photoabsorption is generally much improved. In an exemplary study, Daniels *et al.* (2011 ▸) measured the diffuse scattering of a single crystal having edge lengths of 1.3 × 1 × 1 mm of the relaxor 0.96% Bi_0.5_Na_0.5_TiO_3_–0.04% BaTiO_3_ with 87.6 keV photons at the ESRF with and without an applied electric field, and could observe the changes in numerous Brillouin zones simultaneously (Daniels *et al.*, 2011 ▸, 2012 ▸). Here, we present the determination of the full elastic stiffness tensor of thiourea from TDS measured at photon energies of 100 keV.

**Figure 1:**
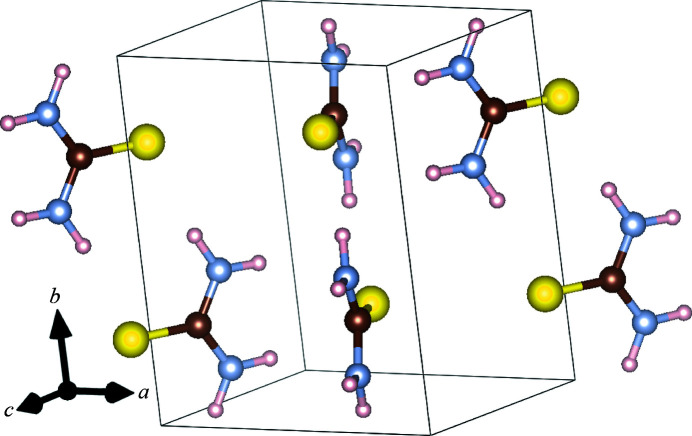
The crystal structure of thiourea under ambient conditions (phase V, *Pnma*) refined on the basis of neutron diffraction intensities (Mullen *et al.*, 1978 ▸). Dark-brown spheres at the centres of the molecules are carbon, large yellow spheres are sulfur, medium-sized pale-blue spheres are nitrogen and small pink spheres are hydrogen. The unit-cell parameters are *a* = 7.657 (4) Å, *b* = 8.588 (5) Å and *c* = 5.485 (3) Å.

**Figure 2:**
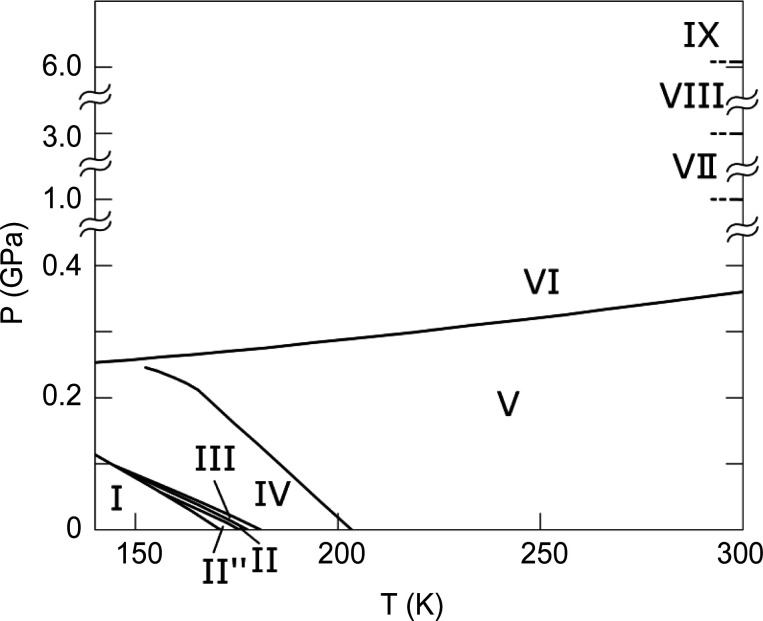
The phase diagram of thiourea. Phase transitions were first reported by Bridgman (1938 ▸) (V → VI), Goldsmith & White (1959 ▸) (I → II, II → III, III → IV and IV → V), Moudden *et al.* (1979 ▸) (phase II′′) and Banerji & Deb (2007 ▸) (VI → VII, VII → VIII and VIII → IX). Phase boundaries were investigated by Klimowski *et al.* (1976 ▸) and Benoit *et al.* (1983 ▸). Phase V crystallizes in *Pnma* (Wyckoff & Corey, 1932 ▸) and phase I in *Pmc*2_1_ (Goldsmith & White, 1959 ▸).

Thiourea, SC(NH_2_)_2_, has received significant attention during the past century since it exhibits some interesting properties. At ambient temperature and pressure, it crystallizes in space group *Pnma* with four formula units per unit cell (Wyckoff & Corey, 1932 ▸) (Fig. 1 ▸), now known as phase V. Thiourea undergoes several phase transitions dependent on both temperature and pressure (Fig. 2 ▸). Goldsmith & White (1959 ▸) conducted measurements of the dielectric coefficient at lower temperatures and found four new phases: phase I below 169 K, phase II between 169 and 176 K, phase III between 176 and 180 K, and phase IV between 180 and 202 K. Later, a phase II′′ was introduced by Moudden *et al.* (1979 ▸). Phases I and III are ferroelectric, and phase I crystallizes in the non-centrosymmetric space group *Pmc*2_1_ (Goldsmith & White, 1959 ▸). Phases II, III and IV have modulated structures. The structural modulation can be described as a sinusoidal transverse wave of atomic shifts propagating along the *c* axis (Shiozaki, 1971 ▸). Thiourea also undergoes a number of pressure-induced phase transitions at 0.34 GPa (Bridgman, 1938 ▸) and 1, 3 and 6.1 GPa (Banerji & Deb, 2007 ▸) (Fig. 2 ▸).

The elastic stiffness coefficients of thiourea were investigated under ambient conditions by Benoit & Chapelle (1974 ▸) and Jakubowski & Ecolivet (1980 ▸) using Brillouin spectroscopy, and by Haussühl & Pähl (1986 ▸) with plane-wave ultrasound spectroscopy (Table 1 ▸). In addition, Haussühl & Pähl (1986 ▸) determined the elastic coefficients for lower temperatures down to 150 K. They confirmed the phase transitions at 202 K (V → IV), 176 K (IV → III) and 169 K (II → I). The longitudinal components of the elastic coefficients determined by Benoit & Chapelle (1974 ▸) and Haussühl & Pähl (1986 ▸) are in very good agreement, while those from Jakubowski & Ecolivet (1980 ▸) are about ten percent higher. The coefficients *c*_23_, *c*_44_ and *c*_66_ agree within less than 1 GPa and *c*_13_ and *c*_55_ agree within about 1.4 GPa for the three previous studies. For the value of *c*_12_, the results of Benoit & Chapelle (1974 ▸) and Haussühl & Pähl (1986 ▸) are in close agreement (∼2.3 GPa) but the value given by Jakubowski & Ecolivet (1980 ▸) deviates by nearly 5 GPa. The bulk moduli (Table 1 ▸) were calculated from the elastic coefficients and they show again that, overall, the results of Benoit & Chapelle (1974 ▸) and Haussühl & Pähl (1986 ▸) are close but those of Jakubowski & Ecolivet (1980 ▸) differ. Since Jakubowski & Ecolivet (1980 ▸) did not state any errors on their results, it is difficult to judge the accuracy of their results with respect to those of the other studies. Overall, it seems that the data set from Haussühl & Pähl (1986 ▸) is the most reliable.

**Figure 3:**
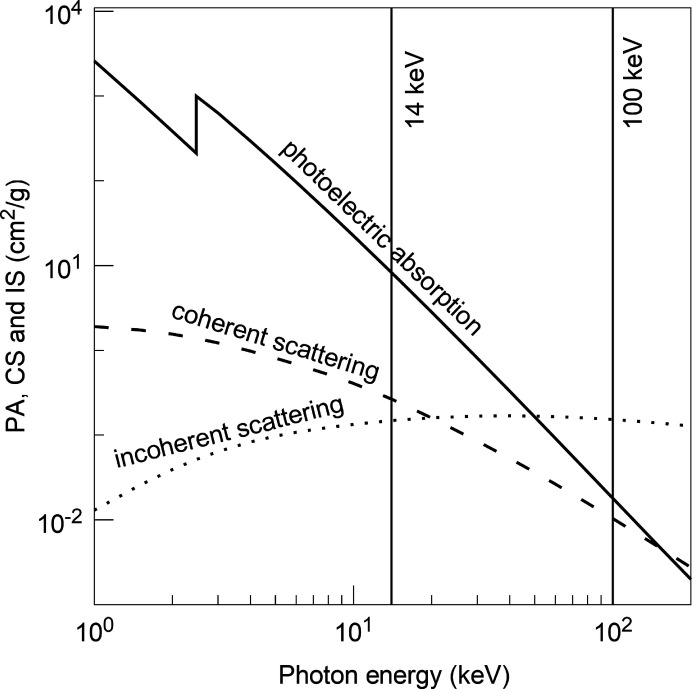
The photoelectric absorption coefficient (PA), coherent scattering (CS) and incoherent scattering (IS) as functions of photon energy calculated for thiourea [data from Berger *et al.* (2010 ▸)].

The reason why previous measurements of organic samples at moderate photon energies resulted in radiation damage is that the photoelectric absorption coefficient is very large at low energies. Here, we make use of the fact that it decreases strongly with increasing photon energy (Fig. 3 ▸). At 100 keV, for thiourea, it is almost three orders of magnitude lower than at 14 keV. At the same energy the coherent scattering decreases by only about one order of magnitude while the incoherent scattering remains nearly constant. The ratio between coherent and incoherent scattering is less favourable at 100 keV, but the advantages at high energies outweigh the disadvantages.

Representative calculations using the program *RADDOSE* (Zeldin *et al.*, 2013 ▸) showed that the dose absorbed by a thiourea single crystal with an edge length of 100 µm is 30 times higher at 14 keV compared with experiments at 100 keV. The dose after which the diffraction intensities of macromolecular crystals are reduced by 30–50% is a few tens of MGy. The intermolecular bonds in thiourea are weak and similar to those in macromolecular crystals. Hence, it can be expected that a dose of a few tens of MGy would make TDS measurements impossible. *RADDOSE* (Zeldin *et al.*, 2013 ▸) calculations show that this dose would be reached in a few minutes at a typical synchrotron beamline at 14 keV, while it will take several hours at 100 keV.

Beamline P21.1 at PETRA III, DESY, has been designed for high-energy measurements and provides photon energies of 52, 85 and 100 keV. Also, the beamline is equipped with a modern hybrid pixel detector, and so the experiments were carried out there.

## 2. Experimental details   

### 2.1. Samples   

**Figure 4:**
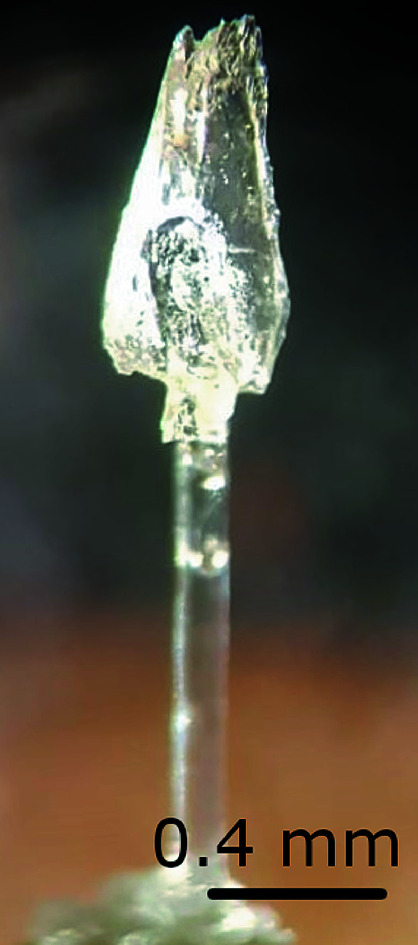
The single crystal of thiourea used for the TDS experiments, mounted on a glass fibre.

We used the same single crystal with dimensions of ∼0.4 × 0.4 × 1 mm (Fig. 4 ▸) for all our measurements. It was grown by S. Haussühl from a solution in methanol at about 315 K using the controlled evaporation technique (Haussühl & Pähl, 1986 ▸).

### 2.2. Experimental setup   

We conducted single-crystal diffraction experiments on beamline P21.1 at PETRA III (https://photon-science.desy.de/facilities/petra_iii/beamlines/p21_swedish_materials_science/p211_broad_band_diffraction/index_eng.html2) at 235, 265 and 295 K using ∼100 keV photons. These temperatures were chosen so as to be convenient. Ambient temperature is a good starting point, and 265 and 235 K can be easily reached by employing a nitrogen jet. The choice of a Δ*T* of 30 K was based on previous studies (Wehinger *et al.*, 2017 ▸; Girard *et al.*, 2019 ▸) where it was used successfully.

In order to fit the elastic stiffness coefficients successfully to the TDS, a high-resolution map of the reciprocal space is required. We therefore used a CdTe PILATUS 1M detector with a pixel size of 0.172 × 0.172 µm, which provides a high dynamic range and allows near noiseless measurements (Dectris Ltd, 2019 ▸). The beam had a size of 500 × 500 µm and a maximum flux of ∼2 × 10^11^ photons per second. There was no special shielding of the background other than that provided by the beamline. The incoming X-rays are directly converted into charge pulses in the CdTe sensor, so that nearly no intensity is spread between neighbouring pixels, and hence the point spread function of this detector is smaller than the pixel size. Thus, it allows the detection of weak TDS close to strong Bragg reflections. At 100 keV, the detector has a quantum efficiency of 56% (Dectris, 2019 ▸). During our experiment, two of the eight panels of the detector were not functional, which caused a loss of data, but, as we will show, this did not prevent successful data analysis.

We employed a nitrogen cryostreamer with a temperature stability of ±2 K to cool the sample. At each temperature, we conducted φ scans where each frame was exposed for 5 s, covering a φ rotation of 0.09° over a total range of 180°.

The distance between the detector and sample was ∼946 mm. This is an important factor in the data collection, as a larger distance corresponds to a higher resolution in reciprocal space. The TDS signal will be registered in a larger number of pixels, thus facilitating the fitting procedure described below. On the other hand, as the detector is fixed, the large distance limits the number of accessible reflections, effectively reducing the number of independent data points. The chosen value is a compromise and is based on experience.

## 3. Computational details   

First-principles calculations were carried out within the framework of density functional theory (DFT) (Hohenberg & Kohn, 1964 ▸), employing the Perdew–Burke–Ernzerhof (PBE) exchange-correlation functional (Perdew *et al.*, 1996 ▸) with a Tkatchenko–Scheffler correction for dispersion forces (Tkatchenko & Scheffler, 2009 ▸) and the plane-wave/pseudopotential approach implemented in the *CASTEP* simulation package (Clark *et al.*, 2005 ▸). ‘On the fly’ norm-conserving pseudopotentials generated using the descriptors in the *CASTEP* database were employed in conjunction with plane waves up to a kinetic energy cutoff of 990 eV. The accuracy of the pseudopotentials is well established (Lejaeghere *et al.*, 2016 ▸). A Monkhorst–Pack (Monkhorst & Pack, 1976 ▸) grid was used for Brillouin-zone integrations with a distance of <0.03 Å^−1^ between grid points. Convergence criteria included an energy change of <5 × 10^−6^ eV per atom for self-consistent cycles, a maximal force of <0.008 eV Å^−1^ and a maximal component of the stress tensor of <0.02 GPa. Phonon frequencies were obtained from density functional perturbation theory calculations.

Calculations for thiourea were carried out to estimate the radiation damage of an isometric crystal of thiourea with edge lengths of 100 µm caused by a beam with 2 × 10^12^ photons per second, where the photons have energies of 14 and 100 keV with an exposure time of 6 min each, using the program *RADDOSE* (Zeldin *et al.*, 2013 ▸). Other input parameters for the software comprise crystallographic information, properties of the beam and details of the geometry of the experiment.

## 4. TDS   

**Figure 5:**
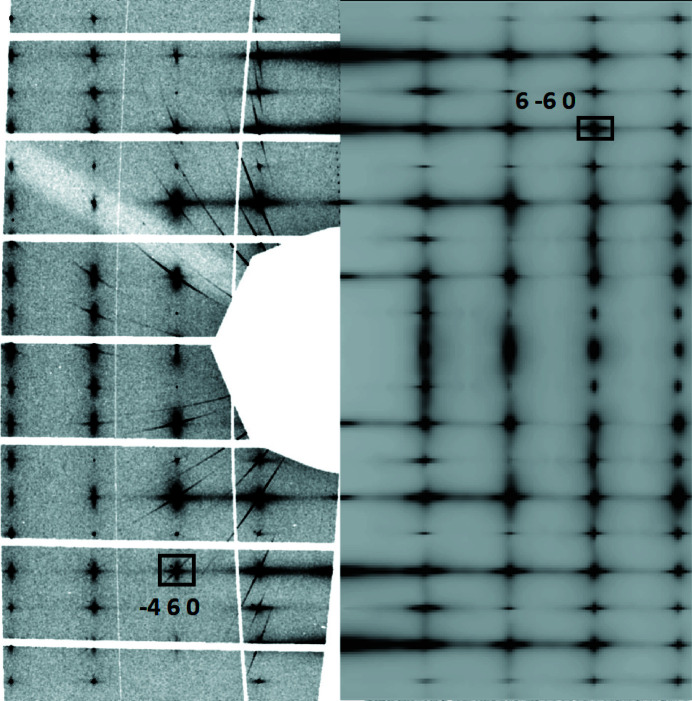
TDS of thiourea in the (*hk*0) plane. (Left) Experimental data for phase V collected at 235 K. (Right) Data generated using *AB2TDS* (Mirone & Wehinger, 2013 ▸) with phonon frequencies and eigenvectors calculated with DFT for phase I in the athermal limit. Since the computed unit-cell parameters of phase I of thiourea differ slightly from the experimentally determined unit-cell parameters of the ambient-temperature phase, the calculated data were scaled by a factor of ∼1.02 in *h* and *k* in order to match the TDS images.

We employed the open-source package *AB2TDS* (Mirone & Wehinger, 2013 ▸) to predict the TDS of thiourea. The program allows the computation of TDS based on phonon frequencies and eigenvectors calculated with atomistic model calculations. Here, we employed results from the DFT calculations described above. The model calculations had to be carried out for phase I, space group *Pmc*2_1_, since phase V (*Pnma*) is experimentally found to be stable only above 202 K, and this leads to dynamic instabilities in calculations restricted to the athermal limit. However, the structural distortions of phase I relative to phase V are very small, and a comparison of the theoretical data with the results of measurements performed at 235 K shows a generally good agreement (Fig. 5 ▸).

## 5. Determination of *c*_*ij*_   

The technique employed here was first presented by Wehinger *et al.* (2017 ▸). It is based on the fit of the elastic stiffness coefficients *c*_*ij*_ to the TDS intensities close to Bragg spots in ‘regions of interest’ (ROIs). In order to fit the intensities successfully, approximate starting values for the *c*_*ij*_ coefficients are required. The ROIs in reciprocal space are chosen so that they are close enough to the reciprocal-lattice points to include diffuse scattering due to acoustic phonons, but far enough from them to ensure that Bragg scattering is excluded. Typical distances from the pixels in which the TDS is analysed to the nearest Bragg spot are ∼0.06–0.15 Å^−1^. Then, mainly acoustic phonons contribute to the TDS intensity. The TDS intensity can be written as 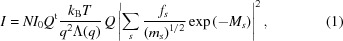
with 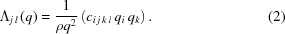

*N* is the number of unit cells, *I*_0_ denotes the incident beam intensity, *Q* represents the total scattering vector, *k*_B_ is the Boltzmann constant, *T* stands for temperature, *q* indicates the momentum transfer, *s* enumerates the atom, *f* symbolizes the atomic scattering factor, *m* is the mass, *M* represents the Debye–Waller factor and ρ is the density (Wehinger, 2013 ▸).

To determine the elastic stiffness coefficients, the equation of motion, 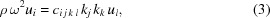
with frequency ω, wavevector **k** = {*k_x_*, *k_y_*, *k_z_*} and displacement vector **u**, has to be solved for the given crystal symmetry (Fedorov, 1968 ▸). The scattering intensities can then be calculated by summing over the three phonon branches in equation (1). The obtained intensities are renormalized by an array *g*(*Q*) regarding absorption, polarization and geometric factors. For background, an array *b*(*Q*) is added. The elastic tensor can be determined with a set of experimental intensities 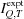 by solving the optimization problem 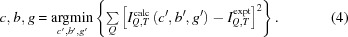
In addition, *c* is constrained by the crystal symmetry and *b* and *g* are kept constant in the vicinity of individual Bragg reflections (Wehinger *et al.*, 2017 ▸). Further mathematical background is provided by *e.g.* Wehinger (2013 ▸), Wehinger *et al.* (2017 ▸) or Girard *et al.* (2019 ▸).

We fitted the TDS data using the open-source package *TDS2EL2* (Mirone & Wehinger, 2017 ▸) with the multi-temperature approach (Wehinger *et al.*, 2017 ▸). This method allows the subtraction of all temperature-independent scattering, including remaining static diffuse scattering that is caused by defects in the crystal structure and surface effects. Diffuse scattering arising from static disorder, air scattering and fluorescence is significantly less dependent on temperature than thermal diffuse scattering. The multi-temperature approach exploits this fact by performing two identical measurements at slightly different temperatures, for instance Δ*T* = 20 K. Then, it is possible to subtract the temperature-independent ‘static’ diffuse scattering (Wehinger *et al.*, 2017 ▸): 
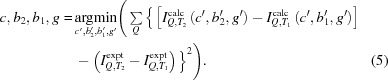



To fit the *c*_*ij*_ using *TDS2EL2*, the first step is to perform a peak search. After identifying the diffraction maxima in the data set, the orientation of the crystal is determined using the graphical user interface included in *TDS2EL2*. Afterwards, the unit-cell parameters (Table 2 ▸) and other geometric parameters, *e.g.* the detector distance and beam position, can be refined and the Bragg reflections can be indexed. The software takes account of symmetry constraints of the crystal provided by the user. When the geometric parameters of the experiment and the crystallographic properties of the crystal have been established, the non-Bragg scattering data around the Bragg spots that will be fitted to the *c*_*ij*_ coefficients are determined in user-defined ROIs. For the multi-temperature approach, two data sets collected at different temperatures are run through this process separately and then combined. For fitting our data, we used the *c*_*ij*_ coefficients obtained by Haussühl & Pähl (1986 ▸) as starting values. Debye–Waller factors were not taken into account for the calculations, since tests showed that they do not impact the results significantly. Instead, default values of 1 were employed.

**Figure 6:**
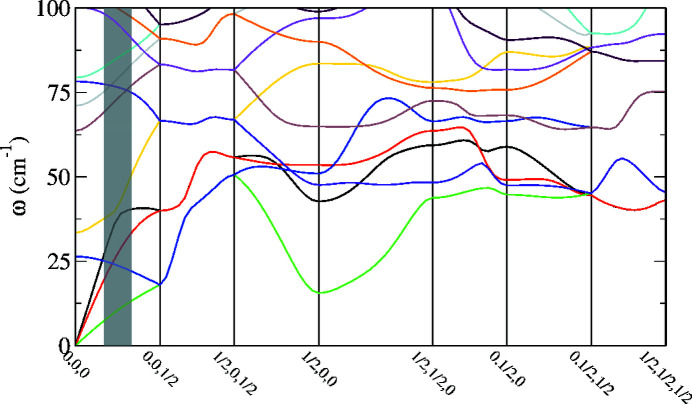
Phonon dispersion relations of thiourea, calculated using DFT. The distance between 0, 0, 0 and 0, 0, ½ is 0.1830 Å^−1^. The overlaid grey rectangle indicates the ROI (0.06–0.12 Å^−1^) used for fitting with *TDS2EL2*.

We subtracted the data set measured at 265 K from the one measured at 295 K since the temperature dependence of the elastic stiffness tensor of thiourea is negligible in this temperature interval (Haussühl & Pähl, 1986 ▸). We fitted the *c*_*ij*_ in the vicinity of 190 intense Bragg spots between *q* = 0.06 Å^−1^ and *q* = 0.12 Å^−1^. The choice of the region employed for the fit was based on the phonon dispersion relations of thiourea (Fig. 6 ▸). We used 0.06 Å^−1^ as a lower limit on the basis of previous studies (Wehinger *et al.*, 2017 ▸; Girard *et al.*, 2019 ▸). We fitted several ROIs between 0.06 and 0.2 Å^−1^ to determine whether this change had noticeable consequences on the results of the fitting procedure. We noted that this was generally not the case, as fits up to 0.2 Å^−1^ resulted in only small changes, although the acoustic phonons are only linear up to 0.095 Å^−1^. The underlying formalism relies on constant slopes of the acoustic phonons, thus restricting the usable range, but on the other hand an extended range simplifies the fitting procedure. Hence we chose, as a compromise, an ROI from 0.06 to 0.12 Å^−1^, which gave the results shown in Table 1 ▸.

**Figure 7:**
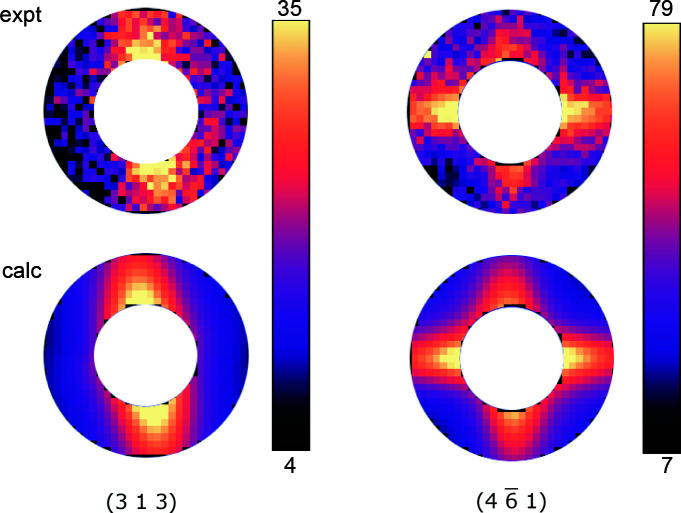
Comparison between (top) experimental TDS and (bottom) calculated TDS of the 313 and 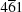 Bragg reflections using *c*_*ij*_ fitted to our experimental data (Table 1 ▸) with an ROI between *q* = 0.06 Å^−1^ and *q* = 0.12 Å^−1^. The intensity scale is logarithmic.

We ensured the precision and robustness of our fit in several ways. Changing one starting value of the *c*_*ij*_ coefficients by 100% does not impede the fit. Further tests showed that, generally, starting values should not differ by more than 15% from the final results to ensure a rapidly converging fit. We graphically examined the quality of the fit by calculating the TDS with our fitted *c*_*ij*_ values using the simulation option in *TDS2EL2*. The reconstructed and experimental TDS are in excellent agreement (Fig. 7 ▸).

## 6. Results and discussion   

As expected from the *RADDOSE* calculations described above, our samples remained intact without any noticeable degradation of the scattering signal after being exposed to the beam for several hours. Although the use of high photon energies leads to an increase in incoherent scattering (Fig. 3 ▸), we did not observe any significant elevation of the background. Since we employed high energies, we were able to increase our sample’s volume so that scattering effects due to surface quality were negligible. Overall, we were successfully able to gather evaluatable TDS intensities for an organic crystal (Fig. 5 ▸).

**Figure 8:**
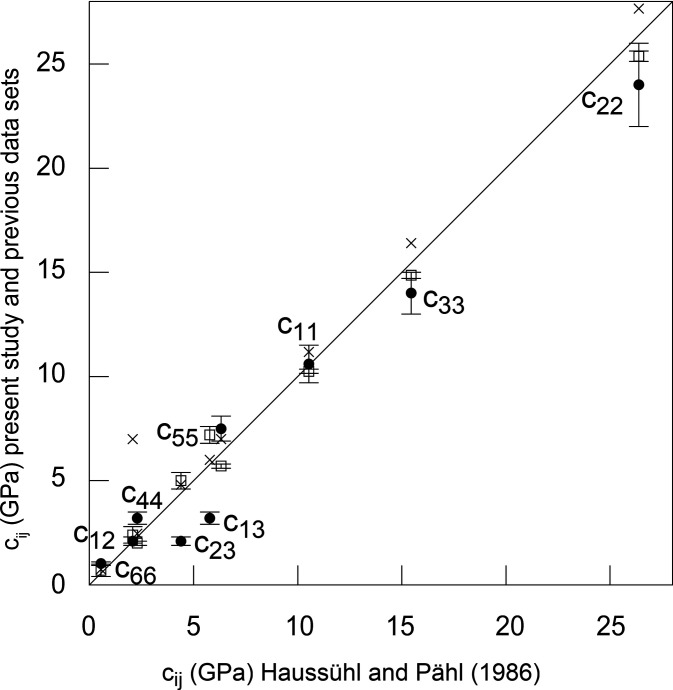
Comparison of data obtained by Haussühl & Pähl (1986 ▸) with ultrasound spectroscopy at 273 K with our data at 280 K (filled circles) and the data sets at ambient temperature from Jakubowski & Ecolivet (1980 ▸) (crosses) and Benoit & Chapelle (1974 ▸) (open squares). The straight line is a guide to the eye representing a perfect correspondence between the reference data and the other data sets.

As benchmarks and initial starting values for the fit we used the *c*_*ij*_ coefficients reported by Haussühl & Pähl (1986 ▸). Their results seem the most reliable because they employed a highly accurate method (improved Schaefer–Bergmann method), they report the smallest errors and their *c*_*ij*_ values agree mostly with the results obtained by Benoit & Chapelle (1974 ▸). Our results are listed in Table 1 ▸ and are shown in Fig. 8 ▸. The overall agreement is very satisfactory, and clearly the TDS-based approach provides data with an accuracy similar to other approaches. Since the elastic coefficients for thiourea are generally small, some relative discrepancies are large, although the absolute discrepancies are small (<3 GPa). When comparing the bulk compressibility, the value calculated from our results [6.5 (5) GPa] is lower than the compressibility calculated from the ultrasound spectroscopic data [7.68 (5) GPa] (Table 1 ▸), but they still agree within 1.2 GPa. This is sufficiently accurate for most applications, such as calculating the velocity of sound waves.

**Figure 9:**
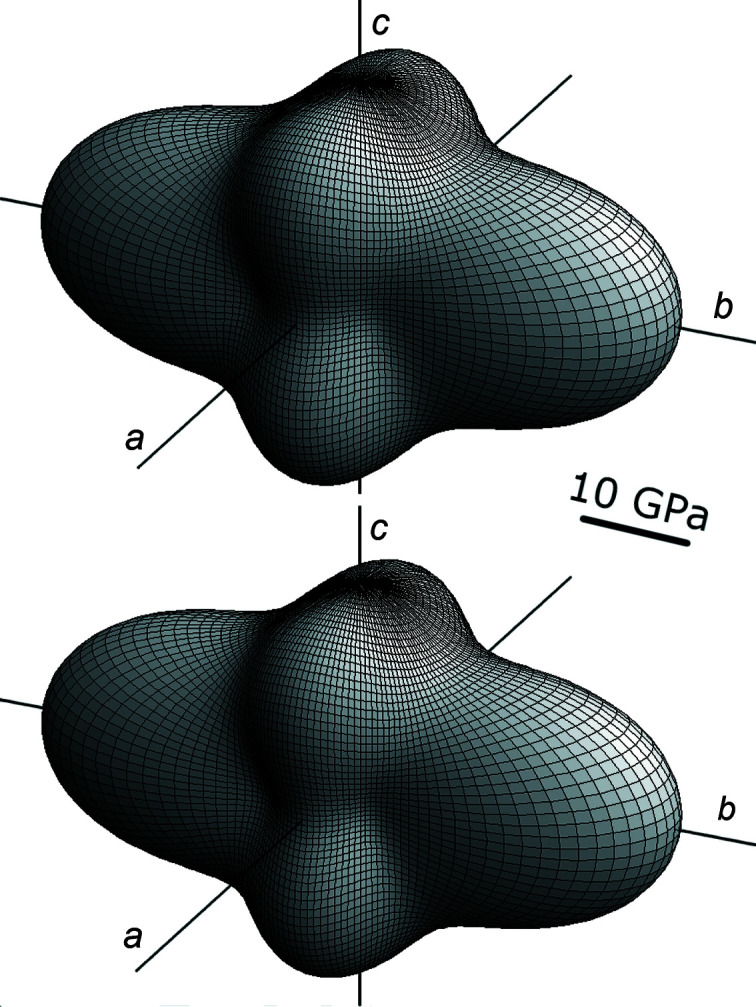
Graphical representations of the longitudinal stiffness of thiourea from (top) our data at 280 K and (bottom) data obtained by Haussühl & Pähl (1986 ▸) at 273 K.

The similarity between the two data sets can be visualized by a comparison of a representation surface for the longitudinal elastic stiffness effect, where we employ a tensor surface defined by the equation 
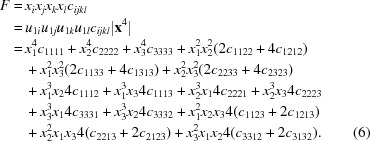
Here *x_i_* = 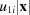 are the components of the radius vector 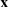, which points from the origin to the tensor surface. *u*_1*i*_ are the cosines of the angle between 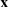 and the Cartesian vector 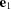 (Arbeck *et al.*, 2010 ▸). The difference between the two representations shown in Fig. 9 ▸ is very small.

## 7. Conclusions and outlook   

We have determined the elastic stiffness tensor from TDS for an organic compound with high-energy photons. The method provides satisfactory agreement with previously published data, and our results show that it is sufficiently accurate for most applications, such as calculating the velocity of sound waves.

The approach may be improved further, *e.g.* by using slightly lower phonon energies (*e.g.* 85 keV). This would lead to an improvement in the signal-to-noise ratio and to shorter measuring times, as for a CdTe-based detector the quantum efficiency would increase from 56% (at 100 keV) to 77% (at 85 keV), while the absorption and hence the radiation damage would probably not be affected. The accuracy could probably also be improved by increasing the number of reflections used in the fit, although this might necessitate a more complex diffraction geometry.

This study has demonstrated the applicability of a TDS-based determination of an elastic stiffness tensor for radiation-sensitive materials, and hence it is now possible to study efficiently compounds such as metal–organic frameworks, for which only small crystals unsuitable for alternative approaches are available. Such measurements are currently underway.

## 

## Figures and Tables

**Table 1:** Elastic stiffness coefficients of thiourea from previous studies and results of this study The *c_ij_* values for TDS are given for 280 K (the average of 295 and 265 K). The errors of the elastic stiffness coefficients obtained by TDS were estimated by running ten fits from different starting values and determining the variation of every *c*_*ij*_. The deviation describes the difference between our coefficients and those of Haussühl & Pähl (1986 ▸) at 273 K. Bulk moduli were calculated from the *c*_*ij*_.

	Benoit & Chapelle (1974 ▸)	Jakubowski & Ecolivet (1980 ▸)	Haussühl & Pähl (1986 ▸)	Haussühl & Pähl (1986 ▸)	Present study	Deviation (%)
Method	Brillouin scattering	Brillouin scattering	Ultrasound spectroscopy	Ultrasound spectroscopy	TDS	
*T* (K)	293	293	293	273	280	
*c*_*ij*_ (GPa)						
*c*_11_	10.25 (10)	11.18	10.24 (3)	10.53 (3)	10.6 (9)	0.7
*c*_12_	2.4 (4)	7.0	2.24 (5)	2.08 (5)	2.1 (2)	1.0
*c*_13_	7.2 (4)	6.0	5.67 (6)	5.78 (7)	3.2 (3)	−44.6
*c*_22_	25.37 (25)	27.66	25.95 (5)	26.38 (6)	24 (2)	−9.0
*c*_23_	5.0 (4)	4.8	4.43 (6)	4.39 (6)	2.1 (2)	−52.2
*c*_33_	14.86 (15)	16.4	15.03 (3)	15.45 (4)	14 (1)	−9.4
*c*_44_	2.0 (1)	2.45	2.22 (2)	2.30 (2)	3.2 (3)	39.1
*c*_55_	5.7 (1)	7.0	6.08 (3)	6.32 (3)	7.5 (6)	18.7
*c*_66_	0.7 (3)	0.74	0.58 (1)	0.55 (1)	1.02 (9)	85.5
Bulk modulus (GPa)	8.0 (3)	9.0	7.56 (5)	7.68 (5)	6.5 (5)	−15.4

**Table 2:** Comparison of unit-cell parameters of thiourea under ambient conditions (phase V) obtained by Tomkowiak & Katrusiak (2018 ▸), by Mullen *et al.* (1978 ▸) and in this study from a refinement of the diffraction data using *TDS2EL2*

Unit-cell parameter (Å)	Tomkowiak & Katrusiak (2018 ▸)	Mullen *et al.* (1978 ▸)	Present study
*a*	7.5791 (9)	7.657 (4)	7.567 (1)
*b*	8.533 (8)	8.588 (5)	8.55 (3)
*c*	5.4655 (3)	5.485 (3)	5.498 (5)
